# Comparison of biochars derived from different types of feedstock and their potential for heavy metal removal in multiple-metal solutions

**DOI:** 10.1038/s41598-019-46234-4

**Published:** 2019-07-08

**Authors:** JingJing Zhao, Xin-Jie Shen, Xavier Domene, Josep-Maria Alcañiz, Xing Liao, Cristina Palet

**Affiliations:** 1 Oil Crops Research Institute of Chinese Academy of Agricultural Sciences/Key Laboratory of Biology and Genetic Improvement of Oil Crops of the Ministry of Agriculture, Wuhan, 430062 China; 2 GTS-UAB Research Group, Department of Chemistry, Facultat de Ciències, Universitat Autònoma de Barcelona, 08193 Cerdanyola del Vallès, Catalunya Spain; 3 Centre for Research on Ecology and Forestry Applications (CREAF), 08193 Cerdanyola del Vallès, Spain; 4 Universitat Autònoma Barcelona, 08193 Cerdanyola del Vallès, Spain

**Keywords:** Environmental sciences, Environmental chemistry

## Abstract

Three different types of feedstocks and their biochars were used to remove Cr(III), Cd(II), Cu(II) and Pb(II) ions from a mixture of multiple heavy metals. The effect of the initial concentration of heavy metals in solution has been analysed, and kinetics modelling and a comparison of the adsorption capacity of such materials have been performed to elucidate the possible adsorption mechanisms. The results show that the adsorption capacity is dependent on the type of feedstock and on the pyrolysis conditions. The adsorption capacity of the biomass types is ranked as follows: FO (from sewage sludge)>> LO > ZO (both from agriculture biomass waste)>> CO (from wood biomass waste). Biochars, which are the product of the pyrolysis of feedstocks, clearly improve the adsorption efficiency in the case of those derived from wood and agricultural biomasses. Complexation and cation exchange have been found to be the two main adsorption mechanisms in systems containing multiple heavy metals, with cation exchange being the most significant. The pore structure of biomass/biochar cannot be neglected when investigating the adsorption mechanism of each material. All the disposal biomasses presented here are good alternatives for heavy metal removal from wastewaters.

## Introduction

Chromium, copper, cadmium and lead are the main heavy metal species in the wastewater industry^1,2^. Relatively modest concentrations of Cr(III), Cd(II) and Pb(II) have toxic effects on the environment and humans. Cu(II) is also a potential toxicant at high doses^3^. According to the World Health Organization (WHO), maximum concentration limits of Cr(III), Cu(II), Cd(II) and Pb(II) (<0.55 mg/L for Cr(III), <0.017 mg/L for Cu(II), <0.01 mg/L for Cd(II), and <0.065 mg/L for Pb) have been established for irrigation water^4–7^. To address heavy metal contamination, biosorption is a promising technique for the removal of contaminants from wastewaters due to its low cost and eco-friendly nature compared with other methods^8,9^. Biosorption processes are based on the use of feedstocks or biomasses, which are usually wastes from agriculture, wood from forests, and sewage industrial sludge^10–12^.

On the other hand, biochar is a porous carbonaceous material obtained during the oxygen-limited pyrolysis of biomass derived from a variety of feedstocks^13^. Biochar has proven to be effective in the removal of heavy metal contaminants from wastewaters due to its specific properties, such as a large surface area, a porous structure, surface-enriched functional groups and the presence of some mineral components^14,15^. The heavy metal adsorption efficiency of biochars can vary widely depending on the types of feedstocks and the pyrolysis temperature^16,17^. The most commonly used feedstock to produce biochar is agricultural waste, such as corn, rice, fruit peels, and wood from forests. In addition, biochar derived from original materials, such as daily manure, wastewater sludges and micro algae, has also been studied in the last decade^18–20^. Therefore, a large body of literature focuses on the use of biochar to remove heavy metals, such as Pb(II), Cu(II), Cr(III), Cd(II), Ni(II) and Zn(II), which are the most studied metals from wastewaters^21,22^.

Biochar has good removal efficiencies in single-metal systems but lower capacities in multiple-metal systems due to the competition between the heavy metals present in wastewaters. Based on the literature, five sorption mechanisms have been proposed to explain biochar adsorption systems, which vary considerably with biochar properties and the target metals. These mechanisms include electrostatic interactions, cation exchange, complexation with functional groups, metal precipitation and reduction of metal species^9,14^. However, few studies have compared the sorption capacities of biochar derived from different types of feedstocks via different sorption mechanisms in multiple-heavy-metal systems. Therefore, it is necessary to study the sorption mechanisms of heavy metals on biochar to improve the metal removal efficiency and guide the application of biochar in the future. Most importantly, biochar application can help solve the large worldwide problem of biomass disposal.

In this study, three types of feedstock from wood, agriculture and industrial sewage sludge wastes were used to remove Cr(III), Cd(II), Cu(II) and Pb(II) ions from multiple-metal systems. Additionally, three biochars were produced from poplar, corn and sewage sludge to determine the influence of the pyrolysis process on the adsorption systems. The objectives of this study are (1) to compare the adsorption capacities of the three different types of feedstocks and derived biochars and (2) to evaluate the possible adsorption mechanisms of biochar in multiple-heavy-metal systems.

## Materials and Methods

### Biomass and biochars

Biomass obtained from different sources, namely, poplar biomass (CO, from wood), sewage sludge (FO, from industry sewage sludge wastes), corn (ZO) and *Brassica napus* (LO) biomasses (both from agriculture wastes), were chosen to evaluate their adsorption capacities, and were all used to remove Cr(III), Cd(II), Cu(II) and Pb(II) ions in multiple-metal systems. Additionally, three biochars (CL, ZL and FL) were produced from poplar, corn and sewage sludge, respectively. These biochars were thermally dried and pyrolysed at the Prat del Llobregat wastewater treatment plant (WWTP) (Barcelona, Spain), and all were produced by slow pyrolysis processes. The temperature conditions and duration of the pyrolysis processes, together with a description of the original biomasses, are listed in Table 1. While poplar, corn, sewage sludge and their biochars were kindly provided by the Centre for Research on Ecology and Forestry Applications (CREAF, Barcelona, Spain), *Brassica napus* is produced in China and was kindly provided by the Oil Crops Research Institute, Chinese Academy of Agricultural Sciences, Wuhan.

**Table 1:** Feedstock biomasses and biochar preparation procedure description.

Materials	Description	Pyrolysis (temperature)	Time (min)
CO	Populus nigra (Poplar) wood		
CL	Biochar of populus	Slow pyrolysis 500–550 °C	15
ZO	Carozo Zea mays		
ZL	Biochar of Carozo	slow pyrolysis 400–500 °C	120
FO	Sewage sludge		
FL	Biochar of Sewage sludge	slow pyrolysis 500–550 °C	15
LO	Brassica napus		

### Chemical and reagents

All the chemicals were analytical grade. A 1,000 mg/L stock solution of a multiple-element system was prepared by dissolving the required amounts of Cr(NO_3_)_3_.9H_2_O, Cu(NO_3_)_2_.3H_2_O, Cd(NO_3_)_2_.4H_2_O and Pb(NO_3_)_2_ (all 99% from Panreac, Barcelona, Spain).

### Characterization of adsorbents

Some physical and chemical properties of biochar including pH of materials, surface area, porosity, surface charge, functional groups, and mineral contents, play an important role in explaining the process of sorption of metals. For this purpose, the morphologies of biomasses and their biochars were analysed by scanning electron microscopy (SEM) at the Electron Microscopy Facilities of the *Universitat Autònoma de Barcelona* (UAB, Catalunya, Spain). Attenuated total reflectance Fourier transform infrared spectroscopy (ATR-FTIR, Tensor 27, Bruker, USA) was performed to identify the chemical functional groups present on the adsorbents. FTIR data were obtained in the wavenumber range of 600 to 4000 cm^−1^ with an average of 16 or 64 scans at 4.0 cm^−1^ resolution at *Servei d’Anàlisi de Química* (UAB, Catalunya, Spain). A Flash 2000 C.E. Elemental Analyzer (Thermo Fisher Scientific, USA) was used to analyse the C and H components of the biochars. A Flash EA 1112 Elemental Analyzer (Thermo Fisher Scientific, USA) was used to analyse N. The O/C, H/C and N/C ratios were calculated from the molar concentrations of the elements of interest, and each ratio was calculated by dividing the total weight of the element by its molecular weight^23^. Brunauere Emmette-Teller technique (BET, TriStar II 3020, Micromeritics, USA) is performed to calculate the surface area and pore structure of materials. The sample is heated at 200 °C for 4 hours under nitrogen vacuum condition for mesoporous measurement. Zeta potential (Zen 3600, Malvern, USA) was performed to indentify the surface charge of materials. Triplicate measurements were performed and each sample was measured 3 times to determine the zeta potential values. Inductively coupled plasma optical emission spectrometry (ICP-OES) using a Varian 725-ES Radial ICP Optical Emission Spectrometer (Varian Inc., USA) was used to analyse K, Ca, Mg and P. The pH of the materials was measured as follows (as reported elsewhere): biochars were prepared in triplicate by adding water at a ratio of 1:10 (biochar/g:deionized water/mL) and vertically agitating for 24 h at a speed of 60 rpm. Then, the suspensions were vacuum filtered with Whatman 42 filter paper, and the pH was measured immediately^23^. The components of the biochars and the pH were analysed at CREAF, and the equipments were all from *Servei d’Anàlisi de Química* (UAB, Catalunya, Spain).

### Batch adsorption experiments

Adsorption experiments were carried out at room temperature (25 ± 1 °C). Multiple-metal solutions (containing Cr(III), Cu(II), Cd(II) and Pb(II)) were prepared from 1,000 ppm initial stock solutions of each metal, and the initial heavy metal concentration ranged from 5 to 100 ppm. Batch experiments were performed by adding 25.00 mg of adsorbent in 5.00 mL tubes and then adding 2.50 mL of heavy metal aqueous solutions, adjusted to pH 4.0. The tubes were then placed on a rotary mixer (CE 2000 ABT-4, SBS Instruments SA, Barcelona, Spain) and shaken at 25 rpm for 24 h. The two phases were separated by decantation and filtered through 0.22 μm Millipore filters (Millex-GS, Millipore). The concentrations of heavy metals in the supernatant phase were analysed by ICP-mass spectrometry (MS) (XSERIES 2 ICP-MS, Thermo Scientific, USA). The adsorption of the selected heavy metals by the adsorbents was expressed as the adsorption percentage calculated by using Eq. (1). Furthermore, the capacity of the adsorbent was calculated by using Eq. (2):

1 $$ \% \,Adsorption=\frac{({C}_{0}-{C}_{e})}{{C}_{0}}\times 100\,$$

2 $${q}_{e}=\frac{({C}_{0}-{C}_{e})\times V\,}{m}$$

where *q*_*e*_ (mg/g) is the capacity of the adsorbent, expressed as the amount of heavy metal per adsorbent mass unit at equilibrium; *V* (L) is the volume of the heavy metal solution; *C*_0_ and *C*_*e*_ are the initial and equilibrium heavy metal concentrations in solution (both in mg/L), respectively; and *m* (g) is the dry weight of the adsorbent. To study the adsorption mechanism, it is more convenient to convert *q*_*e*_ into mmol/g. All the results are expressed as the mean value of duplicate measurements.

## Results and Discussion

### SEM characterization analysis

**Figure 1:**
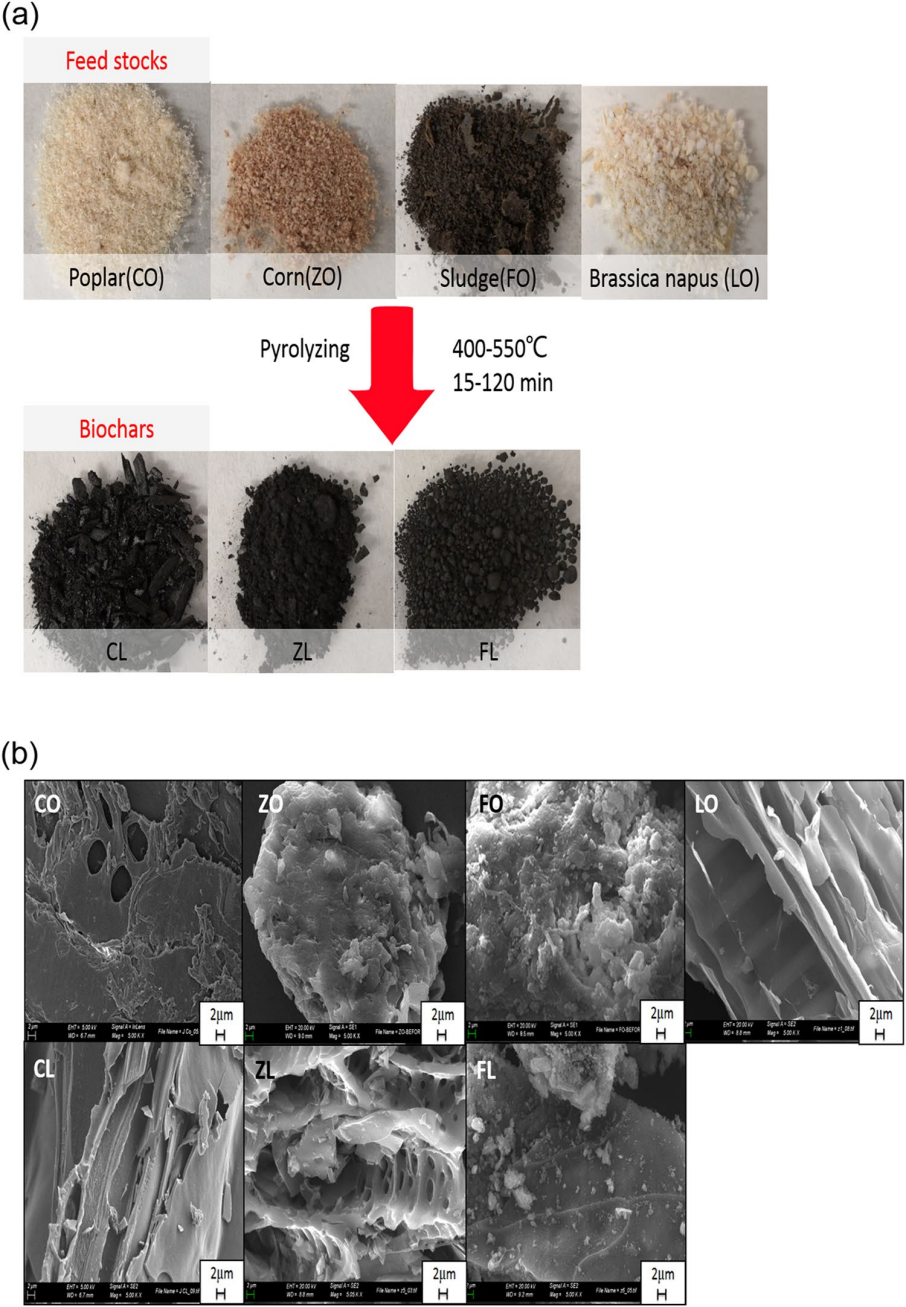
Images of biomasses and biochars. (**a**) Images of raw CO, CL, ZO, ZL, FO, FL and LO. (**b**) SEM images of CO, CL, ZO, ZL, FO, FL and LO.

**Figure 2:**
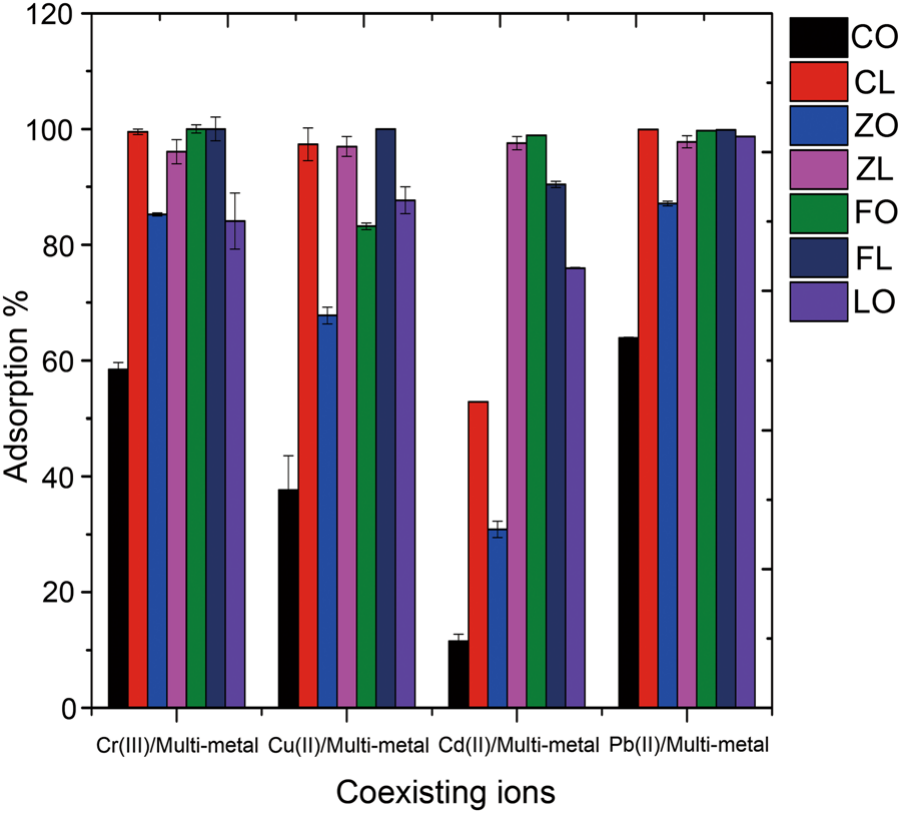
Adsorption of Cr(III), Cd(II), Cu(II), and Pb(II) ions by CO, CL, ZO, ZL, FO, FL and LO in the multiple-metal aqueous system. Initial metal concentration of 0.18 mmol of each metal, contact time of 24 h, initial pH of 4.0, and 25 mg of adsorbent in 2.5 ml of initial solution.

SEM was used to study the morphological structure of the biomass and biochar. As shown in Fig. 1, biochars CL and ZL show more pore structures than CO and ZO, respectively. Furthermore, FL did not have much change in porosity compared with the original biomass FO (see Fig. 1). Additionally, LO has been shown to have a higher heavy metal removal efficiency than CO and ZO (see Fig. 2), which can be explained by the high porosity and the large pore size of LO. This behaviour clearly shows that LO has a pore structure similar to that of CL and ZL, even before pyrolysis (see Fig. 1). Thus, the results presented here demonstrate that pore structure is a key factor that can influence the sorption of heavy metals onto biomass, and Bagreev *et al*. reported similar results^24–26^.

### ATR-FTIR characterization analysis

**Figure 3:**
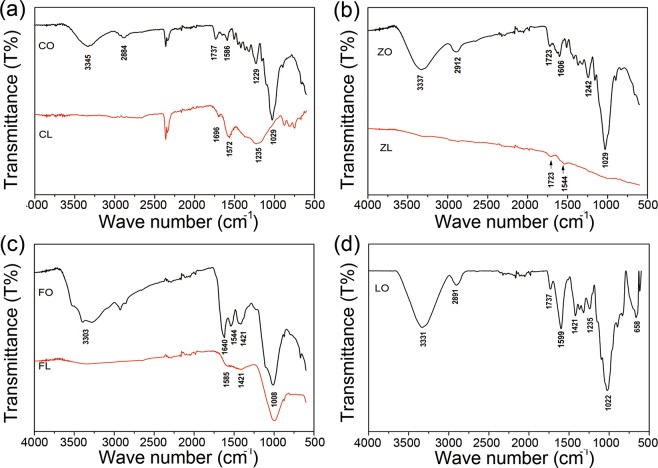
ATR-FTIR spectra of CO and CL (**a**), ZO and ZL (**b**), FO and FL (**c**), and LO (**d**).

ATR-FTIR analysis was carried out to identify the functional groups present in the different adsorbents that might be involved in the sorption process. FTIR spectra of biomass and their biochars are shown in Fig. 3(a–d). The wavenumbers and approximate assignments of the vibrational modes for the FTIR spectra are listed in Table 2. The peaks at 3200–3270 cm^−1^ and 1780–1710 cm^−1^ correspond to the O-H and C=O stretching vibrations, respectively, which confirms the presence of carboxyl groups on the adsorbents^27^. Carboxyl acid groups are very useful for the adsorption of heavy metal ions and can be found in most of the adsorbents studied here (ZO, ZL, CO, CL, LO), except for FO (sewage sludge) and the corresponding biochar FL (see Fig. 3c). These differences can be explained by the compositions of ZO, CO, and LO, which are cellulose and lignin-based biomasses that contain carboxyl groups. However, FO and FL are from industry sewage sludge, and their main components are carbon, hydrogen, oxygen and nitrogen, which are suitable for the production of activated carbon^28^.

**Table 2:** FTIR spectral band assignments for CO, CL, ZO, ZL, FO, FL and LO before use.

Wave numbers (cm^−1^)	Assignments	Sorbents						
		CO	CL	ZO	ZL	FO	FL	LO
3200–3700	O-H stretching	3346		3337		3303		3331
2700–3000	C-H_n_ stretching	2884		2912				2912
1780–1710	Carboxylic Acid C=O stretching	1737	1698	1723	1723			1737
1750–1630	Ketone, Ester, Amide C=O Stretching	1688	1672	1606	1644	1640 1644	1686 1421	1599
1000–1200	C=O/C-O-C	1229	1236	1242				1236
750–870	C-N/R-O-C/R-O-CH_3_ stretching aromatic C-H	1029	1029	1029		1008	1008	1022 868

Furthermore, a decrease in the intensity of the peaks corresponding to carboxyl (–COOH) and hydroxyl (–OH) groups is observed in the FTIR spectra after pyrolysis, probably due to the loss of functional groups in the lignocellulosic materials with increasing temperature. The decrease in the H/C and O/C atomic ratios for biochars (Table 3) confirms this hypothesis. On the other hand, a reduction in the amounts of negative surface charges (related to functional groups such as –COOH, –COH and –OH) will increase the pH of the biochar and, thus, the metal adsorption efficiency of such materials^29^. In this sense, ZL material has higher basicity than CL (measured as explained in the Characterization section, which pH values are shown in Table 3) that can explain also its higher heavy metals adsorption. The adsorption results for these biochars were as follows: LO > ZL > CL (see Fig. 2). The finding that LO has a higher adsorption efficiency than ZL and CL can be explained by the higher amount of carboxyl functional groups on the surface of LO that are available to react with heavy metals (see Fig. 3d).

**Table 3:** Physicochemical properties of CL, ZL and FL.

	CL wood	ZL corn	FL sewage sludge
Components	Cellulose (50%), Hemicellulose (25–35%), Lignin (15–25%)	Cellulose/glucan (37%), Xylan (21%), Lignin (18%)	Carbon (50–70%), Hydrogen (6–7.3%), Oxygen (21–24%), Nitrogen (15–18%)
Temperature	500–550 °C	400–500 °C	500–550 °C
pH	8.2	10.3	8.7 (FO 8.0)
BET surface area m²/g	15.0 (CO 10.8)	22.3 (ZO 17.1)	31.4 (FO 18.5)
Zeta potential (pH 4) mV	−13.6 (CO −2.12)	−32.1 (ZO −0.857)	−5.24 (FO −17.1)
H/C	0.026	0.29 (ZO 1.7)	0.054 (FO 0.17)
O/C	0.15	0.090 (ZO 0.74)	0.22 (FO 2.5)
K g/kg	6.6	23 (ZO 9.4)	9.1 (FO 4.1)
Ca g/kg	9.6	2.6 (ZO 0.22)	89 (FO 41)
Mg g/kg	1.3	1.2 (ZO 0.15)	12 (FO 5.5)
P g/kg	2.0	1.8 (ZO 0.20)	51 (FO 24)

H/C and O/C values are the molar ratio.

Furthermore, BET analysis show that for all biochar systems they have higher surface area values than the corresponding biomass. These results are collected in Table 3. As expected, large surface increase adsorption.

The zeta potential values at pH 4.0 are showed in Table 3. It can be seen that the zeta potential values of biomass (CO, ZO) were more close to zero value than biochars (CL, ZL), which means less negatively charged than biochar. It means that more negative charge on the surface of biochar leading to more chance for electrostatic interactions with heavy metal. The negative charge values at pH 4 ranked as: ZL > FO > CL > FL > CO > LO > ZO. Therefore, the surface charge increased after the pyrolysis process except the case of FL. This behavior can explain the increase on the heavy metal adsorption when using biochar systems.

### Mineral composition analysis

Based on the literature, mineral composition, including potassium (K), calcium (Ca), magnesium (Mg) and phosphorus (P) in biomass and biochar, is also responsible for metal adsorption from aqueous solutions^14,30^. As seen from the results of the mineral composition analysis of all adsorbents under study (collected in Table 3), the mineral concentrations of FO and FL (from sewage sludge) are much higher than those of agriculture waste (ZO, LO) and wood biomass (CO). Furthermore, the concentrations of mineral components (K, Ca, Mg, P) increased after pyrolysis (see Table 3). The pre-concentration of minerals on biochar is mainly due to the formation of biomass ash during pyrolysis. FO and FL have higher mineral concentrations that can provide more opportunities to adsorb heavy metals from water, which can explain the adsorption results (FO > LO > ZO > CO), as shown in Fig. 2. Therefore, this behaviour illustrates the importance of mineral composition in the adsorption process.

The LO biomass yielded promising adsorption results without any modification or pyrolysis process, probably as a result of both its higher porosity level and the important amount of functional groups on its surface, such as carboxyl and hydroxyl groups. Therefore, the authors believe that this material should be studied in more depth in the future.

Next, the adsorption properties of such materials, namely, sewage sludge (FO), wood waste (CO), and agricultural waste (ZO), together with their biochar materials (FL, ZL and CL) will be presented. Followed with the kinetic modelling and the influence of the initial heavy metal concentrations in solution.

### Comparison of biosorbent adsorption properties

As indicated in the previous section, four feedstocks and three biochars were used to remove Cr(III), Cd(II), Cu(II) and Pb(II) ions in multiple-metal systems: biomass CO (poplar from wood), FO (sewage sludge from solid industrial waste), ZO (corn from agriculture waste), and LO (*Brassica napus* from agriculture waste). Additionally, biochars obtained by the pyrolysis of CO, FO and ZO (CL, FL and ZL, respectively) were evaluated as heavy metal adsorbents. These seven sorbents show different biosorption capacities for the different metal ions, as shown in Fig. 2. In general, for all metals, biochars have better sorption capacity than the original biomass, which can be explained by surface changes during the pyrolysis process, such as changes in porosity, functional groups and mineral content. Based on the literature, high pyrolysis temperatures lead to increased porosity and surface area compared with the original biomaterial (as shown in Fig. 1 and Table 3). High porosity and large surface areas can increase the adsorption of metals^31^. High temperature also increases the concentration of minerals (K, Ca, Mg and P) on the surface of sorbents that can be used for ion exchange with heavy metals^31–33^. Minerals from biomass are not burned, so the pyrolysis process acts as a mineral pre-concentration step. The adsorption percentages according to the type of feedstock were ranked as follows: sewage sludge (FO) >> agriculture waste biomass (LO) > (ZO) >> wood biomass (CO). This ranking can be explained by the different mineral compositions and functional groups present, which is confirmed by the measured adsorption capacity (see Table 3, Fig. 3 and part of the effect of the initial concentration).

### Effect of contact time

**Figure 4:**
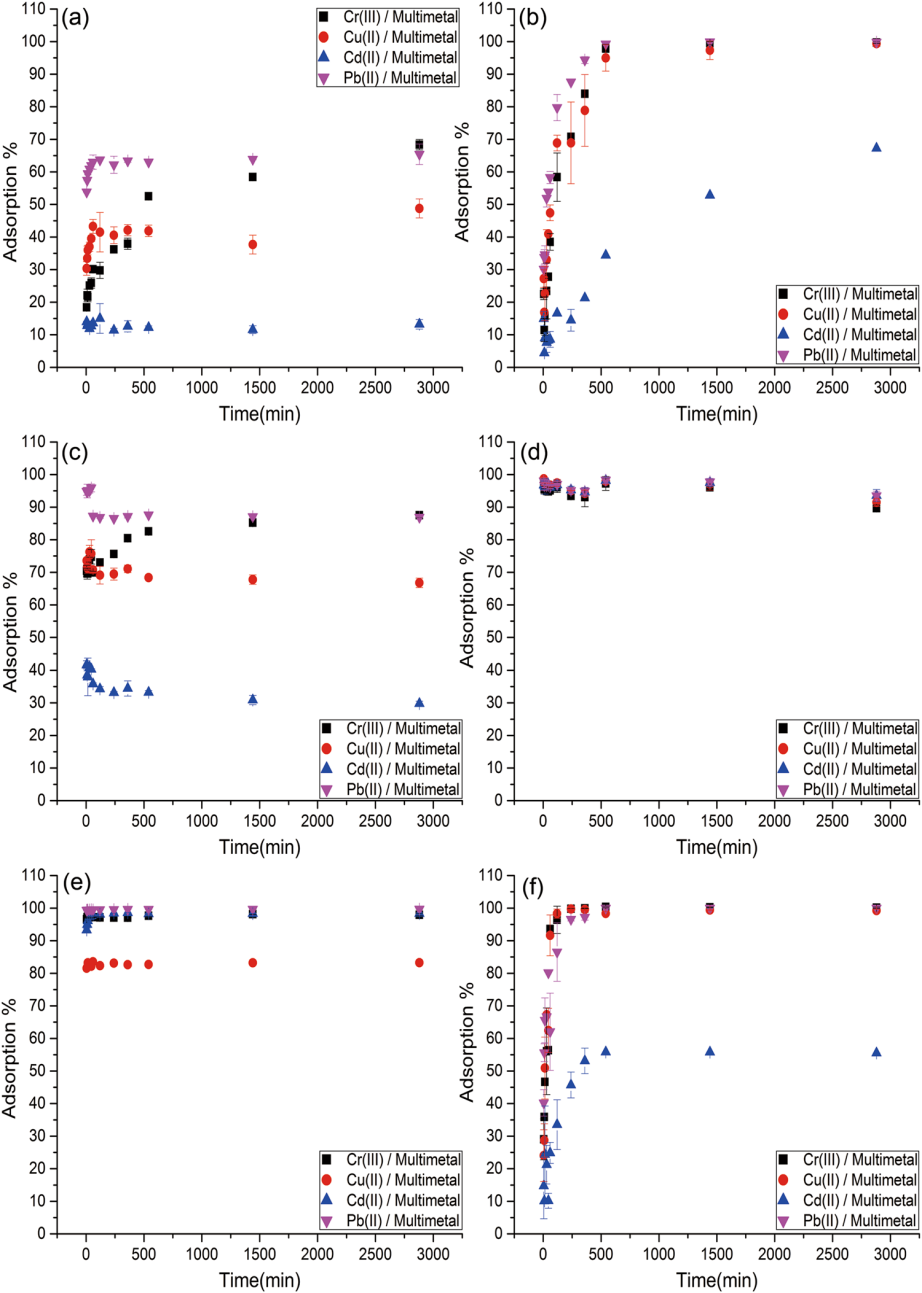
Adsorption percentage of Cr(III), Cu(II), Cd(II), and Pb(II) by CO (**a**), CL (**b**), ZO (**c**), ZL (**d**), FO (**e**) and FL (**f**) from the multiple-metal system at different contact times. Initial metal ions concentration of 0.18 mmol/L of metal, pH 4.0, and 25 mg of adsorbent.

The effect of the contact time between sorbents (CO, CL, ZO, ZL, FO, FL) and heavy metals in multiple-metal systems (Cr(III), Cu(II), Cd(II), Pb(II)) was studied. For that purpose, adsorption experiments were performed (as indicated in the experimental section) for different times (5, 15, 30, 45, 60, 120, 240, 360, 540, 1440 and 2880 minutes) for each adsorbent (Fig. 4). In general, biochars from ZO (from agriculture) wastes were more effective than those from sewage sludge and wood biomass. Adsorption equilibrium was reached at different times for each biomass or biochar. ZO and ZL were both effective at adsorption of all metal ions and reached equilibrium in 5 minutes. In the case of CO, the equilibrium time differed as a function of the heavy metal, so adsorption equilibrium was reached in 5 minutes for Pb(II) and Cd(II), in 1 h for Cu(II), and in 24 h for Cr(III). Additionally, CL reached equilibrium slowly compared with CO, requiring approximately 8 h for Cr(III), Cu(II) and Pb(II) and 24 h for Cd(II). In contrast, biochar from sewage sludge (FL) was less effective than FO (especially for Cd(II)). FL also needed a longer time than FO to reach adsorption equilibrium (approximately 6 h), while FO adsorption of all metals took only 5 minutes. Thus, 24 h was chosen as the optimal contact time for further adsorption experiments.

To understand the different adsorption behaviours in multiple-heavy-metal systems, kinetic analysis was performed to find a model that explains the obtained results and to obtain information about the mechanisms of heavy metal adsorption onto biomass and biochar systems.

### Kinetic modelling

Two different kinetic models, the pseudo-first-order (PFO) and pseudo-second-order (PSO) models, have been widely used to describe adsorption. The PFO and PSO models assume that the rate of metals adsorbed on the surface of sorbents is proportional to the number of unoccupied sites; PFO kinetics is controlled by the physical process, and PSO kinetics is controlled by chemical processes, including valence forces sharing or exchanging electrons between the adsorbent and adsorbate. The PFO and PSO mathematic model expressions are given in Eq. (3) and Eq. (4):

3 $$\mathrm{log}({q}_{e}-{q}_{t})=\,\mathrm{log}({q}_{e})-\frac{{k}_{1}}{2.303}t$$

4 $$\frac{1}{{q}_{t}}=(\frac{1}{{k}_{2}{q}_{e}^{2}})\times \frac{1}{t}+\frac{1}{{q}_{e}}$$

where *q*_*t*_ and *q*_*e*_ are the capacity at time *t* and at equilibrium, respectively (and expressed as mmol/g), and *k*_1_ and *k*_2_ are the rate constants. In most cases, the PFO equation is linear only over approximately the first 30 minutes; therefore, it is appropriate for the initial contact time but not for the whole range^34^. Kinetics modelling analysis showed that the adsorption process did not fit well with the PFO model but fit well with the PSO model for all the adsorbents. This result means that the adsorption of heavy metals on the surface of the adsorbents is a chemical adsorption process, such as valence forces sharing or exchanging electrons between the adsorbent and adsorbate. The relative constants found by applying the model are listed in Table 4 only for Pb in the multiple-metal system. Higher capacities of Pb(II) have been found except in the case of CO.

**Table 4:** Adsorption kinetic constants for the adsorption of Pb(II) by CO, CL, ZO, ZL, FO and FL in the multiple-metal system.

Pb(II)/Multi-metal	Pseudo-second-order model		
	k_2_(g/μmol/min)	q_2_ (μmol/g)	R^2^
CO	0.98	9.20	1.000
CL	0.26	14.7	0.998
ZO	12.0	12.7	1.000
ZL	0.26	16.7	0.999
FO	5.68	16.7	0.999
FL	0.66	16.5	0.999

### Effect of initial concentration

**Figure 5:**
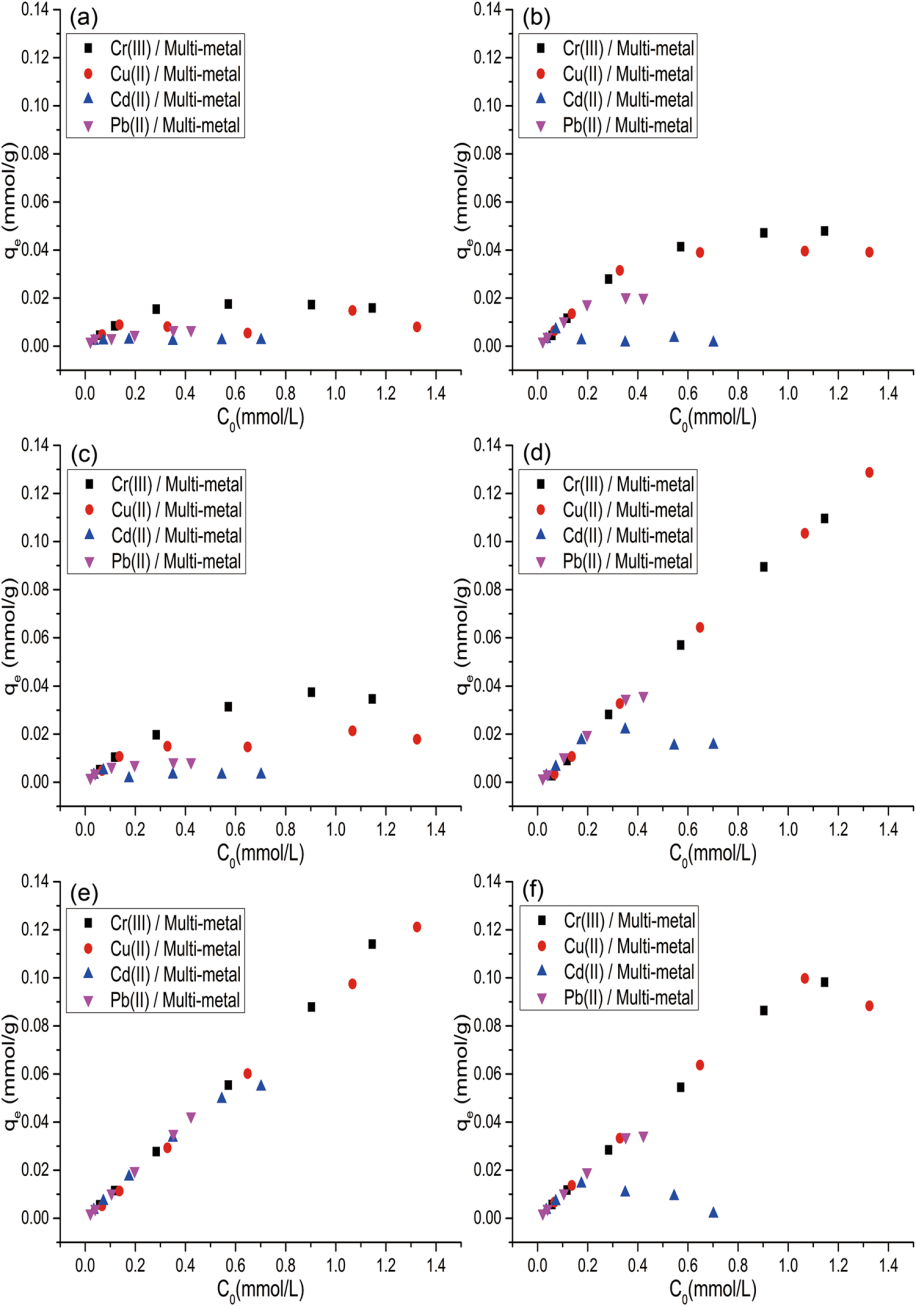
Effect of the initial concentration of heavy metals on their adsorption by CO (**a**), CL (**b**), ZO (**c**), ZL (**d**), FO (**e**) and FL (**f**) in multiple-metal systems. Experimental conditions were T = 25 ± 1 °C, pH 4.0, 25 mg of adsorbent, 2.5 mL of metals solution and stirring for 24 hours.

Five multiple-metal solutions (5 ppm, 25 ppm, 50 ppm, 75 ppm and 100 ppm) were prepared from 1,000 ppm stock solutions of each heavy metal. The initial concentration study provides a significant understanding of the competition between the four heavy metals during the adsorption process. The adsorption capacity of all the adsorbents for the four metals is shown in Fig. 5, and the adsorption capacity for Pb(II) in multiple-metal systems is listed in Table 5. As shown in Fig. 5, the adsorption capacity of heavy metals (Cr(III), Cu(II), Cd(II), Pb(II)) from different types of feedstocks is ranked as follows: FO (from sewage sludge) > CO (from wood) and ZO (from agriculture). All adsorbents have the same priority for heavy metals, such as Cr(III), Cu(II) and Pb(II) metal ions, and the adsorption of Cd(II) was much lower than that of the other metals, probably due to the competition between the heavy metals.

**Table 5:** Adsorption capacity of biomass and biochar in multiple-heavy-metal systems.

q_e_(μmol/g)	CO	CL	ZO	ZL	FO	FL
Cr(III)	15.9	27.9	35.7	109	114	98.2
Cu(II)	9.10	31.7	13.4	128	88.3	99.7
Cd(II)	2.32	2.39	3.02	15.5	54.7	14.3
Pb(II)	6.75	17.5	8.39	35.9	35.1	33.7

The ranking of the adsorption of biochars from different types of feedstocks was ZL (from agriculture) > FL (from sewage sludge) > CL (from wood), which could be explained by the different mineral compositions of the adsorbents. High mineral amounts provide more possibilities for the exchange of heavy metals from the solution (because the concentration of minerals is increased after pyrolysis), which could increase the adsorption capacity. As shown in Table 3, the concentrations of some mineral components of FL (Ca 89.1 g/kg, P 51.2 g/kg) are much higher than those of ZL (Ca 2.55 g/kg, P 1.83 g/kg) and CL (Ca 9.60 g/kg, P 2.00 g/kg), but potassium contents are the exception (FL (K 9.10 g/kg), ZL (K 23.4 g/kg), and CL (K 6.60 g/kg)). This behaviour can be related to the adsorption capacity values of these biochars (see Table 5).

FL has a higher mineral content than FO; however, a slight decrease in the adsorption capacity for Cr(III) and Pb(II) and a much higher decrease in the adsorption capacity for Cd(II) were observed for FL. The slight decrease in FL capacity could be explained by the loss of functional groups on the surface of the biomass. As shown in Fig. 3, most of the functional groups of FL were lost during the pyrolysis process, which was confirmed by the decrease in the H/C and C/H ratios (Table 3).

### Possible adsorption mechanism

In previous studies reported in the literature, five mechanisms have been proposed to govern metal sorption by biochar from aqueous solutions, namely, complexation, cation exchange, precipitation, electrostatic interactions, and chemical reduction^9,14^. However, the role that each mechanism plays for each metal varies considerably depending on the target metals and adsorbents. Fei *et al*. described the molecular-level adsorption of Pb(II) and Cu(II) to peat biomass mainly through carboxyl groups (–COOH)^35^. Whereas both electrostatic interactions and complexation with biochar surfaces are responsible for Cr adsorption and reduction^36,37^.

Until now, few studies have focused on the comparison of different types of feedstocks for the removal of heavy metals from multiple-metal aqueous systems (more similar to real water situations). According to the results of Lu *et al*., the adsorption of Pb(II) by sewage sludge-derived biochar mainly occurred through proton-active carboxyl (–COOH) and hydroxyl (–OH) functional groups on the biochar surface, as well as coprecipitation or complexation on the mineral surfaces^38^. After comparison of the characterization and evaluation of the adsorption capacity of different types of feedstocks, a new perspective has been found to explain the adsorption mechanisms onto biomass and biochar adsorbents. We demonstrate that complexation and cation exchange are the two main adsorption mechanisms, with the influence of cation exchange being larger than that of complexation in the present cases.

Furthermore, an increase in mineral concentrations was found in biochars after the pyrolysis of the corresponding biomasses, which could explain the increase in the adsorption capacity to heavy metals of the biochars (CL and ZL, respectively)^38^. FO and FL (which have higher mineral concentrations than the other adsorbents presented here and lack carboxyl groups on their surfaces) have higher adsorption capacities than CO and ZO (which have lower mineral concentrations and more carboxyl groups). The adsorption capacities of FO and FL are similar, which may be due to the similar porosity of both materials before and after pyrolysis. Although there is an increase in the mineral concentrations on the surface of FL (which will increase its adsorption capacity), the loss of carboxyl groups as a function of temperature during pyrolysis can globally reduce the adsorption capacity of FL for heavy metals^14^. Therefore, FO and FL have similar adsorption capacities.

**Figure 6:**
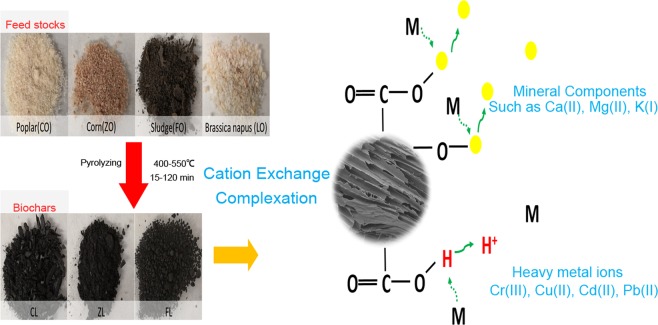
Schematic diagram illustrating the mechanism of heavy metal removal by biomass and/or biochar.

In summary, heavy metals are adsorbed on the surface of biomass/biochar via exchange mainly with Ca, K, and Mg but also with protons from carboxyl and hydroxyl groups. In addition, if these latter functional groups are present at high amounts on the bioadsorbent surface, they can also complex heavy metals from the aqueous solutions (see Fig. 6). Finally, it is important to note that the amount of either mineral or carboxyl groups can differ depending on the composition of the original biomass and, in the case of biochars, as a function of the pyrolysis conditions employed^39,40^.

## Conclusions

Seven biosorbents have been used successfully for the removal of Cr(III), Cu(II), Cd(II) and Pb(II) from multiple-metal aqueous systems. The biochars produced from wood and agriculture wastes have higher adsorption capacities than the initial biomasses do. This finding can be explained by an increase in porosity and a pre-concentration of mineral components during the pyrolysis process. Complexation and cation exchange probably are the two main adsorption mechanisms in multiple-heavy-metal systems, and these mechanisms are influenced by the kind of feedstock and its mineral composition and by the pyrolysis treatment, being more effective for agriculture waste than for wood biomass. The sludge can be used directly to remove heavy metals without pyrolysis pretreatment.

In summary, these disposal biomasses can be used to remove heavy metals from multiple-metal aqueous systems because of their low cost, eco-friendliness and availability. Therefore, biomass could be an interesting alternative to synthetic materials for heavy metal removal. Biochar can also be an alternative, even though it requires biomass pretreatment.
